# Cell Cycle Changes, DNA Ploidy, and PTTG1 Gene Expression in HTLV-1 Patients

**DOI:** 10.3389/fmicb.2020.01778

**Published:** 2020-07-24

**Authors:** Debora Levy, Mari Cleia M. R. Ferreira, Cadiele O. Reichert, Lis Vilela de Almeida, Graciela Brocardo, Luis Alberto P. C. Lage, Hebert F. Culler, Youko Nukui, Sergio P. Bydlowski, Juliana Pereira

**Affiliations:** ^1^Lipids, Oxidation and Cell Biology Team, Laboratory of Immunology (LIM19), School of Medicine, Heart Institute (InCor), University of São Paulo, São Paulo, Brazil; ^2^Department of Hematology, Hemotherapy and Cell Therapy, School of Medicine, University of São Paulo, São Paulo, Brazil; ^3^Pro-Sangue Foundation, Department of Hematology, Hemotherapy and Cell Therapy, School of Medicine, University of São Paulo, São Paulo, Brazil; ^4^Laboratory of Medical Investigation on Pathogenesis and Targeted Therapy in Onco-Immuno-Hematology (LIM-31), School of Medicine, University of São Paulo, São Paulo, Brazil

**Keywords:** HTLV-1, ATL, PTTG1, cell cycle, T-cell lymphocytes

## Abstract

Human T-cell lymphotropic virus type-1 (HTLV-1) is a pathogenic retrovirus that is associated with adult T-cell leukemia/lymphoma (ATL). Genetic instability is the hallmark of ATL. Cell cycle progression is needed for virus particle reproduction. HTLV-1 encoded Tax protein ultimately disrupts the mitotic spindle checkpoint, leading to incorrect chromosome segregation, resulting in aneuploidy. Cell cycle abnormalities have been described in T cells transfected with HTLV-1 virus *in vitro*, but not in HTLV-1 asymptomatic carriers. PTTG1 and HTLV-1 viral protein Tax exhibit a cooperative transforming activity. Overexpressed PTTG1 results in chromosome instability and aneuploidy, which has been suggested as a mechanism underlying PTTG1 transforming activity. Here we aimed to investigate cell cycle, DNA ploidy and PTTG1 mRNA expression in CD4^+^ and CD8^+^ T cells in healthy subjects (HS), HTLV-1 asymptomatic carriers and ATL patients. We have identified that HTLV-1 asymptomatic carriers have shown DNA aneuploidy and cell cycle arrest at cell cycle phase G_0_/G_1_ in CD4^+^ T cells. CD8^+^ T cells of HTLV-1 asymptomatic carriers also demonstrated DNA aneuploidy but without alteration in cell cycle. In ATL, CD4^+^ and CD8^+^ T cells present a higher number of cells in cell cycle S-phase and PTTG1 overexpression. These studies provide insight into malignant transformation of HTLV-1 asymptomatic carriers to ATL patients.

## Introduction

Adult T-cell leukemia/lymphoma (ATL) is an aggressive and incurable malignancy of mature CD3^+^, CD4^+^, CD25^+^, CD7^–^ T cells caused by human T-cell lymphotropic virus type-1 (HTLV-1), which was initially described in 1977 by Takatsuki (2005). HTLV-1 is transmitted perinatally by breastfeeding, by sexual intercourse, and parentally by blood transfusion, injection drug abuse, and transplantation (Yoshizumi et al., 2012). Globally, at least 5–10 million people are infected with HTLV-1 (Gessain and Cassar, 2012). However, the prevalence varies according to the geographic area, with predominance in southwestern Japan, sub-Saharan Africa, Melanesia, the Caribbean, and Central and South America. In Brazil, almost 800,000 people are infected with HTLV-1 (Carneiro-Proietti et al., 2002). Although most infected individuals remain asymptomatic, 2–6% of HTLV-1 asymptomatic carriers (HTLV-1 infected) develop ATL or HTLV-1-associated myelopathy/tropical spastic paraparesis (Verdonck et al., 2007). Generally, ATL occurs after a long period of latency, as long as 20–30 years, but the host- or virus-related factors that influence ATL progression are unknown. The low rate of progression and long latency period suggest that secondary genetic events are important in the oncogenesis of ATL (Marriott and Semmes, 2005).

Significant progress in recent years in ATL research has provided a better understanding of disease progression, with a growing information on the cellular and molecular characteristics of ATL cells (Taylor and Matsuoka, 2005; Watanabe, 2017). Aneuploidy is the hallmark of ATL and is caused by mutations in several genes involved in DNA replication, chromosome segregation, and cell cycle checkpoints (Kasai et al., 2002; Merling et al., 2007; Potapova et al., 2013). HTLV-1 basic leucine zipper factor (HBZ) – which has been described as an important oncogenic component, acts by inducing the proliferation of HTLV-1-infected cells and inhibiting its apoptosis (Mitobe et al., 2015). Besides HBZ, Tax protein is also able to promote genomic instability in HTLV-1-infected cells. Studies have demonstrated that Tax protein interferes in the spindle checkpoint through inhibition of the pituitary tumor transforming 1 gene (PTTG1) (Liu et al., 2003). This oncogene is located on chromosome 5q33 and has been found in pituitary tumors from rats (Pei and Melmed, 1997). PTTG1 blocks the activity of the separase protein to hinder premature chromosome separation during cell division. However, appropriate degradation of PTTG1 by the anaphase-promoting complex/cyclosome (APC/C) is critical to initiating anaphase. After degradation, the separase is released and becomes active, disrupting the cohesion between the chromosomes and allowing accurate segregation of the sister chromatids (Auner et al., 2004). PTTG1 may also block p53 and regulate DNA repair mechanisms and apoptosis (Hornig et al., 2002).

Our group has assessed a cohort of HTLV-1 asymptomatic carriers with the goal of finding clinical and molecular aspects related to ATL progression (Sanabani et al., 2012; Pessoa et al., 2014; de Souza et al., 2018). Herein, we aimed to survey cell cycle, DNA content and PTTG1 gene expression in individual populations of CD4^+^ and CD8^+^ T cells in HTLV-1 infected and ATL patients in comparison to healthy subjects (HS).

## Materials and Methods

### Patients

This study was approved by the local ethics committee (CAPPesq HC-FMUSP number 814/08) and written informed consent was obtained from each participant. A total of 30 mL of peripheral blood was taken from 38 HTLV-1 infected, 20 ATL patients, and 35 healthy subjects. HBV, HCV, HIV, and HTLV-2 serology were performed at diagnosis and all the participants were tested negative. HTLV-1 positivity was confirmed by Western blotting in all HTLV-1 infected and ATL cases included in this study. The diagnosis and ATL classification was carried out according to the World Health Organization and Shimoyama criteria (Shimoyama, 1991). CD4^+^ and CD8^+^ T cells were determined immediately after sample collection. Purity tests, cell cycle and aneuploidy analysis were performed on the same day of blood collection. The samples were kept in Trizol (Invitrogen Corporation, Carlsbad, CA, United States) at −20°C, RNA was extracted and cDNA was synthesized in up to 48 h. The cDNA was aliquoted and stored at −80°C until real-time PCR was performed.

### Sample Preparation and Cell Isolation

Peripheral blood mononuclear cells (PBMCs) were recovered using Ficoll-HyPaque Plus (GE Healthcare Bio-Sciences, United Kingdom) according to the manufacturer’s instructions. Afterward, CD4^+^ and CD8^+^ T cells were isolated by positive selection using an LS magnetic column and anti-human CD8 (isotype mouse IgG2a) and CD4 (isotype mouse IgG1) monoclonal antibodies and microbeads from Miltenyi Biotec (Bergich Gladbach, Germany) following the manufacturer’s protocol. Subsequently, to verify the percentage of CD4^+^ and CD8^+^ T cells obtained in each sample, 1 × 10^6^ cells were stained using the CD4FITC/CD8PE/CD3PerCP panel of monoclonal antibodies (Becton Dickinson, San Jose, CA; clones SK3, SK1, and SK7) and analyzed on a FACS Calibur^®^ using CellQuestPro software (Becton Dickinson, San Jose, CA, United States). Samples with >90% CD4^+^ or CD8^+^ T cells were selected for the next experiments.

### Cell Cycle and DNA Measurement by Flow Cytometry

**FIGURE 1:**
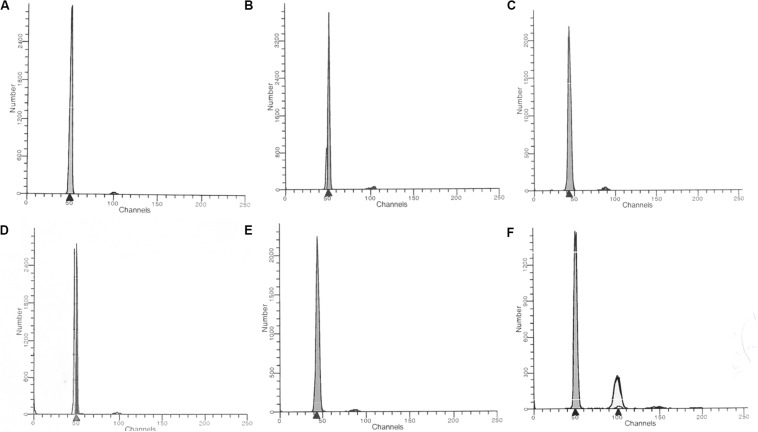
Unidimensional histogram in linear scale obtained by flow cytometry. **(A)** healthy control; **(B)** HTLV-1 infected hyperdiploidy; **(C)** HTLV-1 infected hypodiploidy; **(D)** ATL hyperdiploidy; **(E)** ATL hypodiploidy; **(F)** ATL hyperdiploidy (tetraploidy).

Cell cycle analysis was performed using a commercial DNA kit from Becton Dickinson (San Jose, CA) as indicated. Briefly, 5 × 10^5^ CD4^+^ and CD8^+^ T cells were added to different tubes and stained with propidium iodide (PI; Becton Dickinson, San Jose, CA, United States). Instrument setup and calibration was performed with Calibrite beads (Becton Dickinson, San Jose, CA, United States) and FACSComp^TM^ software version 2.0 (Becton Dickinson, San Jose, CA, United States). The voltage of the canal was adjusted to the mean peak of the G_0_/G_1_ population at 10,000 marks on the histogram plot. The optical performance of the flow cytometer was monitored using control DNA QC (Becton Dickinson, San Jose, CA, United States) according to the manufacturer’s recommendations. The data were analyzed using ModFit LT software version 2.0 (Verity Software House Inc., Topsham, United States) (Figure 1). Histogram was not acceptable if its variation coefficient (CV) (breadth of the fluorescence signal for normal cell population in G_0_/G_1_ phase at the middle of its height) exceeded 8%.

The DNA index ploidy (DI) was determined as the mean channel number of G_0_/G_1_ peak of the diploid or aneuploidy DNA population of the unknown sample, divided by the mean channel of the G_0_/G_1_ diploid peak of the control cells (healthy individual). All data, including DNA index ploidy was provided by the ModFit LT software version 2.0. The DNA index ploidy of the reference cells (diploid cells) used as control was considered 1.0, and a DI <0.95 (hypodiploid) or >1.05 (hyperdiploid) was considered as “DNA aneuploidy.” The presence of at least two separate G_0_/G_1_ peaks was interpreted as true aneuploidy.

### Real-Time Quantitative PCR

Total RNA was isolated from CD4^+^ and CD8^+^ T cells of 38 HTLV-1 infected, 20 ATL patients, and 25 healthy subjects using Trizol reagent (Invitrogen Corporation, Carlsbad, CA, United States) as described by the manufacturer. RNA was measured in a spectrophotometer (Nanodrop, Corbett, United States) and the samples were standardized at a concentration of 1000 ng of RNA. Subsequently 1 μg of RNA was treated with DNase (Promega Corporation, Madison, United States) and the total RNA was converted into cDNA by reverse transcription (Applied Biosystems). Oligonucleotides (primers and probes) specific for PTTG1 mRNA were designed according to Auner et al. (2004): sense primer, 5′GAAGACTGTTAAAGCAAAAAGCTCTGTA 3′; antisense primer, 5′ GGCAGGTCAAAACTCTCAAAGTCTA 3′; probe, 5′ CCTGCCTCAGATGATGCCTATCCAGAAA-Q.

Amplification was performed in a final volume of 12.5 μL. In brief, reactions were carried out using 3 μL cDNA, 1× TaqMan Universal PCR Master Mix (Applied Biosystems, cod 4304437), 0.3 μM of sense and antisense primer, and 0.15 μM of probe. Amplification was performed at 50°C for 20 s followed by a denaturation phase at 95°C for 10 min and 45 cycles of amplification at 95°C for 15 s and 60°C for 1 min. The mRNA expression was normalized using the beta glucuronidase (GUSB) gene (Applied Biosystems, cod. 433767F). The amplification efficiency of each pair of primers was confirmed using standard curves of known RNA concentrations. The KG1 cell line was used as a calibrator and positive control. RNA transcription was measured using 7500 Fast real time PCR system (Applied Biosystems). The assays were carried out in duplicate and the data was collected by the 7500 software v2.0.5 (Applied Biosystems). The expression levels of the target genes were calculated using the Livak and Schmittgen (2001) method.

### Statistical Analysis

The Shapiro–Wilk test was used to determine the characteristics of data distribution. Results were shown as median ± standard deviation (SD). Student’s *T*-test and the non-parametric Kruskal-Wallis test were used to compare the quantitative variables between the groups. Mann-Whitney *U* test non-parametric was used to determine the relation between aneuploidy and PTTG1 expression. To evaluate the association between cell cycle phases and expression of PTTG1 expression, Pearson and/or Spearman’s rank test was performed. Multivariate linear regression was used to establish the relationship between cell cycle and PTTG1 expression.

The multivariate linear regression analysis was carried out through the analysis of stepped models, in which the criteria of choice for the independent variable to be part of the equation was *p* < 0.05 in all stages of the process, as well as Goodness of Fit and the coefficient of determination. Cox proportional hazards ratio (HR) estimation by partial maximum likelihood was used to analyze the interaction between PTTG1 CD4^+^ and CD8^+^ T cells expression with age, gender, DHL, lymphocyte count, lymphocyte percentage and calcium. Statistical analysis was performed in SPSS^®^ for Windows (SPSS Inc., Version 26.0), and a *p* ≤ 0.05 was considered significant.

## Results

### Clinical Characteristics

The individuals were prospectively analyzed from July 2009 to December 2012. The healthy subjects median age was 50 years (24–80 years), 55.5 years for HTLV-1 infected (33–80 years), and 53.5 years for ATL patients (24–72 years). ATL subtypes and clinical aspects are displayed in Table 1.

**TABLE 1:** Clinical findings of casuistic.

	HS (*n* = 35)	HTLV-1 infected (*n* = 38)	ATL (*n* = 20)	*p*
Age, years (median ± SD)	50.0 ± 12.78	55.5 ± 11.34	53.5 ± 13.01	0.269
**Sex, n (%)**				
Male, n (%)	16 (45.7)	14 (36.8)	10 (50.0)	0.568
Female n (%)	19 (54.3)	24 (63.2)	10 (50.0)	
**Laboratory**				
Lymphocytes	N/A	2.0 × 10^9^/L	1.9 × 10^9^/L	0.945
LDH (U/L)	N/A	347.5	623.5	**0.003**
Calcium (median/mg/dL)	N/A	9.5	9.1	NS
**ATL subtypes**				
Acute, n (%)			4 (15.8)	
Chronic, n (%)			6 (31.6)	
Lymphoma, n (%)			5 (26.3)	
Smoldering, n (%)			5 (26.3)	

*p-values were obtained using the Kruskal-Wallis test. HS, healthy subjects; HTLV-1 infected, HTLV-1 asymptomatic carrier; ATL, adult T-cell leukemia/lymphoma; LDH, lactate dehydrogenase; N/A, not available. Normal range: Lymphocytes (0.93–4 × 10^9^/L); LDH (240–480 U/L): calcium: 8.6–10.2 mg/dL.*

### Cell Cycle

HTLV-1 infected showed higher amount of CD4^+^ T cells in phase G_0_/G_1_, with a median of 98.32% (92.98–100%; DP 2.18) in comparison to healthy subjects with a median of 97.14% (86.98–100%; DP 2.96), *p* = 0.041 and ATL patients with a median of 97.25% (91.40–98.32%; DP 3.55), *p* = 0.023. No significant difference was found between ATL and healthy subjects (*p* = 0.575).

The percentage of CD4^+^ T cells in S-phase was significantly higher in ATL, with a median of 1.8% (0.0–10.36%; DP 3.37) in healthy subjects with a median of 0.63% (0.0–7.63%; DP 1.78), *p* = 0.020 and HTLV-1 infected with a median of 0.34% (0.0–6.98%; DP 1.27), *p* < 0.001. HTLV-1 infected and healthy subjects did not show any significant difference in the percentage of cells in S-phase (*p* = 0.073). The median percentage of G_2_/M was not significantly different among the three groups in the CD4^+^ T cells population (*p* = 0.960) (Table 2).

**TABLE 2:** Tlymhocytes cell cycle.

	CD4^+^ T cells				CD8^+^ T cells			
		
G_0/_G_1_ (%)	HS (*n* = 35)	HTLV-1 infected (*n* = 38)	ATL (*n* = 20)	*p*	HS (*n* = 35)	HTLV-1 infected (*n* = 38)	ATL (*n* = 20)	*P*
Median ± SD	97.14 ± 3.0	98.32 ± 2.2	97.25 ± 3.6	0.035*	97.9 ± 2.0	98.3 ± 1.2	97.4 ± 1.9	0.138
Min–Max	86.9–100	93.0–100	89.6–100		91.5–100	94.0–100	92.8–100	
**G_2_/M (%)**								
Median ± SD	0.56 ± 2.9	0.88 ± 1.8	0.99 ± 2.8	0.960	1.0 ± 1.9	0.8 ± 1.2	0.8 ± 1.1	0.374
Min–Max	0.0–13.1	0.0–7.0	0.0–10.4		0.0–8.5	0.0–5.5	0.0–3.3	
**S-phase (%)**								
Median ± SD	0.63 ± 1.8	0.34 ± 1.2	1.80 ± 3.4	0.007**	0.4 ± 1.3	0.5 ± 0.9	1.5 ± 1.7	0.003***
Min–Max	0.0–7.6	0.0–7.0	0.0–10.4		0.0–6.9	0.0–3.7	0.0–6.1	

SD, Standard deviation; HS, Health subjects; HTLV-1 infected, HTLV-1 asymptomatic carriers; ATL, Adult T-cell leukemia/lymphoma. p-value when using the Kruskall-Wallis Test for comparison among the groups. p < 0.05 = * Cells in G_0_/G_*1*_ phase were significantly higher in the HTLV-1 group in CD4^+^ T cells. ** Cells in S-phase were significantly higher in the ATL group in CD4^+^ T cell. *** Cells in S-phase were significantly higher in the ATL group in CD8^+^ T cells.

**FIGURE 2:**
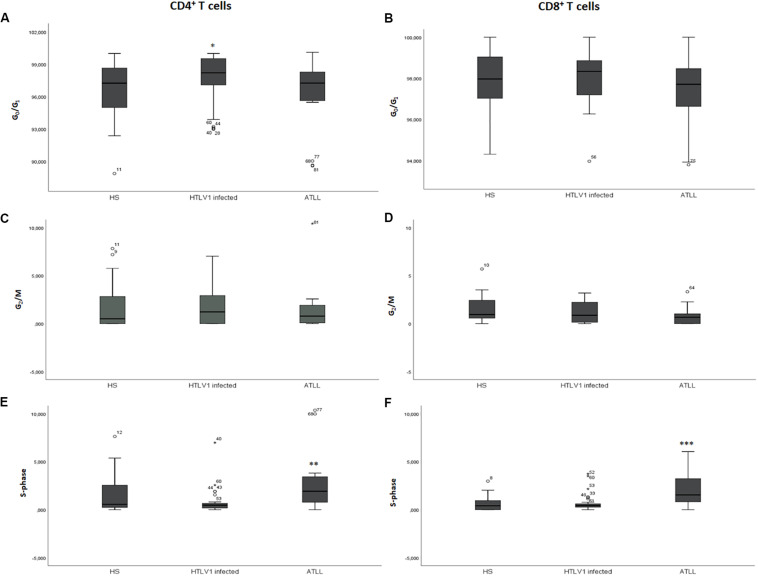
Cell cycle analysis of CD4^+^ and CD8^+^ T cells in the different phases of cell cycle. **(A,C,E)** CD4^+^ T cells; **(B,D,F)** CD8^+^ T cells. A–B: percentage of cells in G_0_/G_1_ phase; **(C,D)** percentage of cells in S-phase; **(E,F)** percentage of cells in G_2_/M phase. ^∗^Cells in G_0_/G_1_ phase were significantly higher in the HTLV-1 infected group in CD4^+^ T cells *p* = 0.035. ^∗∗^The cells in S-phase were significantly higher in the ATL group in CD4^+^ T cells *p* = 0.003. ^∗∗∗^The cells in S-phase were significantly higher in the ATL group in CD8^+^ T cells *p* = 0.003. HS, Health subjects; HTLV-1 infected, HTLV-1 Asymptomatic carriers; ATL, Adult T-cell leukemia/lymphoma. The data represents the median, the minimum and maximum value of the cell cycle phase; between the minimum and maximum values, is the interquartile range.

ATL patients showed higher percentage of S-phase with a median of 1.54%, while HTLV-1 infected showed a median of 0.45% (0–3.73%; DP 0.87) (*p* = 0.003) and healthy subjects a median of 0.41% (0–6.87%; DP 1.30), *p* = 0.001 in CD8^+^ T cells. There was no significant difference between HTLV-1 infected and healthy subjects (*p* = 0.712) in CD8^+^ T cells. The median G_0_/G_1_ was 97.85% (94.31–100%) in healthy subjects, 98.34% (96.34–100%) in HTLV-1 infected, and 97.41% (92.76–100%) in ATL patients (*p* = 0.138) in CD8^+^ T cells. There was no significant difference of G_2_/M percentage among healthy subjects (0.95%); HTLV-1 infected (0.83%) and ATL (0.83%); *p* = 0.374 (Table 2 and Figure 2).

**FIGURE 3:**
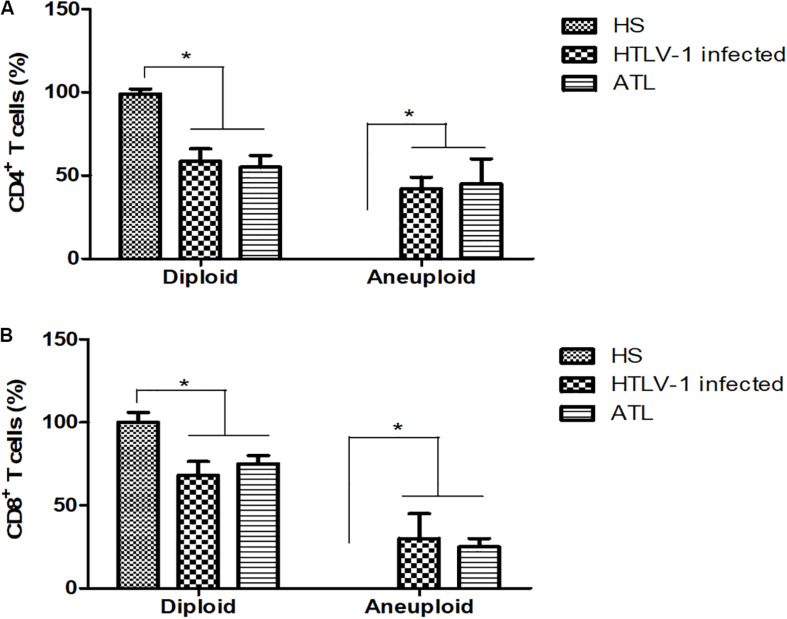
Percentage of CD4^+^ and CD8^+^ T cells with diploid and aneuploidy cells. **(A)** CD4^+^ T cells; **(B)** CD8^+^ T cells. HS, Health subjects; HTLV-1 infected, HTLV-1 Asymptomatic carriers; ATL, Adult T-cell leukemia/lymphoma. *P < 0.005.

The presence of DNA aneuploidy was analyzed in the different groups. None of the healthy subjects presented aneuploidy in CD4^+^ or CD8^+^ T cells. HTLV-1 infected presented DNA aneuploidy in 42.1 and 31.6% of patients in CD4^+^ and CD8^+^ T cells, respectively. In ATL, 45.0% of patients presented DNA aneuploidy in CD4^+^ T cells and 25.0% in CD8^+^ T cells (Figure 3).

### PTTG1 Gene Expression

**FIGURE 4:**
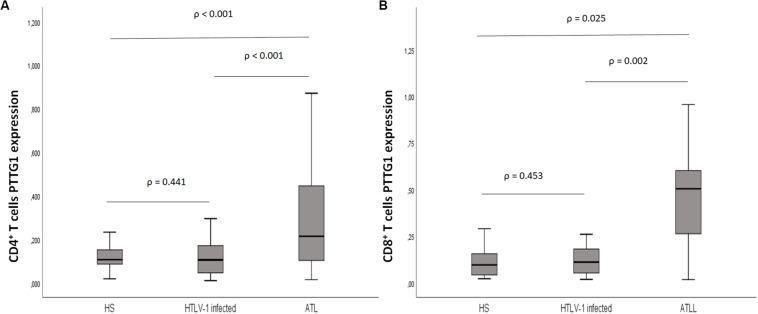
PTTG1 gene expression of T lymphocyte CD4^+^ and CD8^+^ T cells. **(A)** CD4^+^ T cells; **(B)** CD8^+^ T cells. Distribution pattern of the median intensity of gene expression between health subjects, HTLV-1 asymptomatic carriers and ATL. The data represents the median, the minimum and maximum value of the PTTG1 expression; between the minimum and maximum values, is the interquartile range. HS, Health subjects; HTLV-1 infected, HTLV-1 asymptomatic carriers; ATL, Adult T-cell leukemia/lymphoma.

Eighty-three samples were available for PTTG1 analysis, including 25 healthy subjects with median age of 49.6 years (24–80); 38 HTLV-1 infected, median age 55 years (33–80); and 20 ATL, median age 55.3 years (24–72). The median PTTG1 expression in CD4^+^ T cells was higher in ATL (median 0.230, 0.015–1.073; DP 0.294) than in HTLV-1 infected (median 0.105, 0.010–0.662; DP 0.156), *p* < 0.001 and healthy subjects (median 0.106, 0.019–0.310; DP 0.254), *p* = 0.017. No difference was found between HTLV-1 infected and healthy subjects (*p* = 0.441). In CD8^+^ T cells, the median PTTG1 expression was also higher in ATL in comparison to HTLV-1 infected (0.507 vs. 0.106; *p* = 0.002) and healthy subjects (0.507 vs. 0.096; *p* < 0.025). No difference was found between HTLV-1 infected and healthy subjects (*p* = 0.453) (Figure 4).

### PTTG1 Gene Expression and Cell Cycle

The correlation between PTTG1 gene expression and cell cycle was available for 25 healthy subjects, 38 HTLV-1 infected, and 20 ATL patients with the linear regression model. No correlation was observed between PTTG1 expression in the CD4^+^ T cells and the percentage of cells in G_0_/G_1_ for HTLV-1 infected (*R*^2^ = 0.157; *p* = 0.180) and healthy subjects (*R*^2^ = 0.058; *p* = 0.244). On the other hand, a positive correlation was found between ATLL PTTG1 expression in CD4^+^ T cells and the percentage of cells in G_0_/G_1_ (*R*^2^ = 0.302; *p* = 0.018). No correlation was observed between PTTG1 expression in CD4^+^ T cells and the percentage of cells in G_2_/M for HTLV-1 infected (*R*^2^ = 0.158; *p* = 0.640) and healthy subjects (*R*^2^ = 0.066; *p* = 0.472). A positive correlation of ATL PTTG1 expression in CD4^+^ T cells and the percentage of cells in G_2_/M was found (*R*^2^ = 0.362; *p* = 0.034). There was no significant difference between PTTG1 expression in CD4^+^ T cells and the percentage of cells in S-phase (Table 3).

**TABLE 3:** Relationship between PTTG1 expression in CD4^+^ T cells, CD8^+^ T cells, and cell cycle phase.

			G_0_/G_1_			G_2_/M			S-phase		
					
Parameters	Median ± SD	min–max	β Value	Standard error	Model^a^	β Value	Standard error	Model^b^	β Value	Standard error	Model^c^
**PTTG1 expression in CD4^+^ T cells**											
HS	0.106 ± 0.254	0.019–0.310	−0.242	0.252	*R*^2^ = 0.058; *p* = 0.244	−0.127	0.256	*R*^2^ = 0.066; *p* = 0.472	−0.751	0.262	*R*^2^ = 0.066; *p* = 0.687
HTLV-1 infected	0.105 ± 0.156	0.010-−0.662	−0.396	0.143	*R*^2^ = 0.157; *p* = 0.180	−0.029	0.145	*R*^2^ = 0.158; *p* = 0.640	1.566	0.132	*R*^2^ = 0.320; *p* = 0.070
ATLL	0.230 ± 0.294	0.015–1.073	−0.549	0.300	*R*^2^ = 0.302; *p* = 0.018	−0.285	0.296	*R*^2^ = 0.362; *p* = 0.034	−0.419	0.303	*R*^2^ = 0.377; *p* = 0.077
**PTTG1 expression in CD8^+^ T cells**											
HS	0.096 ± 0.083	0.020-−0.290	0.029	0.084	*R*^2^ = 0.010; *p* = 0.892	0.100	0.086	*R*^2^ = 0.003; *p* = 0.962	0.180	0.088	*R*^2^ = 0.005; *p* = 0.992
HTLV-1 infected	0.111 ± 0.182	0.020-−0.910	−0.120	0.179	*R*^2^ = 0.014; *p* = 0.485	0.480	0.181	*R*^2^ = 0.016; *p* = 0.766	0.39	0.184	*R*^2^ = 0.016; *p* = 0.911
ATLL	0.544 ± 0.365	0.020–1.390	0.678	0.267	*R*^2^ = 0.424; *p* = 0.003	−0.349	0.245	*R*^2^ = 0.516; *p* = 0.002	0.193	0.254	*R*^2^ = 0.482; *p* = 0.009

*^*a*^Multivariate linear regression by cell cycle phase G_0_/G_1_; ^*b*^Multivariate linear regression by cell cycle phase G_2_/M; ^*c*^Multivariate linear regression by cell cycle S-phase.*

No association was observed between PTTG1 expression in CD8^+^ T cells and the percentage of cells in G_0_/G_1_ for HTLV-1 infected (*R*^2^ = 0.014; *p* = 0.485) and healthy subjects (*R*^2^ = 0.010; *p* = 0.892). However, a positive correlation of ATL PTTG1 expression in CD8^+^ T cells and the percentage of cells in G_0_/G_1_ was observed (*R*^2^ = 0.424; *p* = 0.003). No correlation was observed between PTTG1 expression in CD8^+^ T cells and the percentage of cells in G_2_/M for HTLV-1 infected (*R*^2^ = 0.016; *p* = 0.766) and healthy subjects (*R*^2^ = 0.003; *p* = 0.962). A positive correlation of ATL PTTG1 expression in the CD8^+^ T cells and the percentage of cells in G_2_/M was found (*R*^2^ = 0.516; *p* = 0.002). No correlation was observed between PTTG1 expression in CD8^+^ T cells and the percentage of cells in S-phase for HTLV-1 infected (*R*^2^ = 0.016; *p* = 0.911) and healthy subjects (*R*^2^ = 0.005; *p* = 0.992). A positive correlation was found between ATL PTTG1 expression in CD8^+^ T cells and the percentage of cells in S-phase (*R*^2^ = 0.482; *p* = 0.009) (Table 3).

### PTTG1 Gene Expression With Aneuploidy Cases and Clinical Features

**FIGURE 5:**
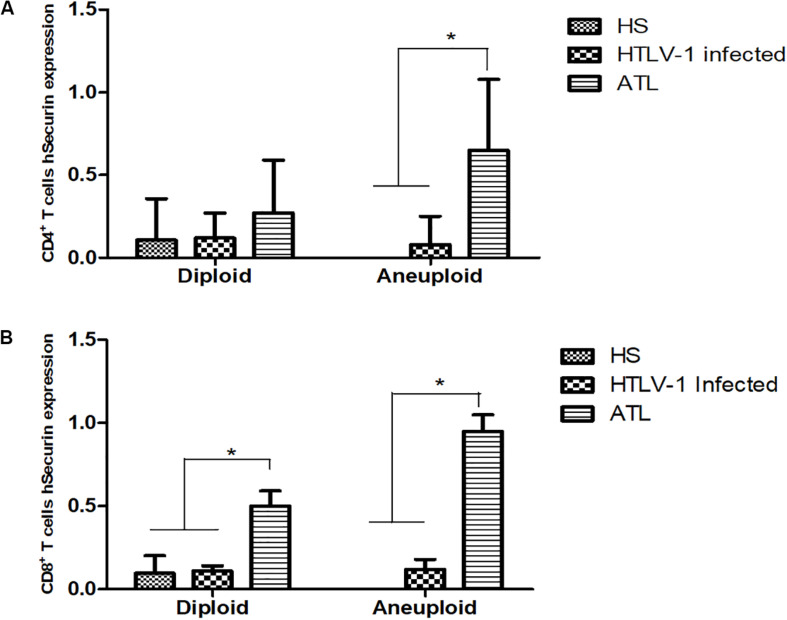
PTTG1 gene expression in diploid and aneuploidy cases. **(A)** CD4^+^ T cells; **(B)** CD8^+^ T cells. HS, Health subjects; HTLV-1 infected, HTLV-1 asymptomatic carriers; ATL, Adult T-cell leukemia/lymphoma. *p < 0.005.

The expression of PTTG1 was analyzed in diploid and aneuploidy cases in the different groups. CD4^+^ T cells presented a PTTG1 expression of 0.107 (±0.06) in the diploid cases without presence of aneuploidy. HTLV-1 infected cases showed no difference in PTTG1 expression (median expression of 0.12 ± 0.15 in diploid cases and 0.08 ± 0.17 in aneuploidy cases). In ATL, aneuploidy cases showed increased PTTG1 expression (0.65 ± 0.43), while diploid cases showed no significant difference (0.27 ± 0.32) (Figure 5A). CD8^+^ T cells presented a PTTG1 expression of 0.096 (±0.10) in the diploid cases. HTLV-1 infected cells showed no difference in PTTG1 expression (median expression in diploid cases was 0.11 ± 0.03 and 0.12 ± 0.06 in aneuploidy cases).

In ATL, both diploid (0.50 ± 0.09) and aneuploidy cases (0.95 ± 0.10) showed increased PTTG1 expression (Figure 5B). Cox regression association between PTGG1 expression in CD4^+^ and CD8^+^ T cells demonstrated that PTTG1 expression in CD4^+^ T cells increased death risk in 18.941 (95%CI 1.286–278.932; *p* = 0.032), while CD8^+^ T cells showed no association (death risk 5.581 95%CI 0.941–33.083; *p* = 0.058) (Table 4).

**TABLE 4:** Cox regression of stratified data in ATL group.

	PTTG1 expression CD4^+^ T cells				PTTG1 expression CD8^+^ T cells			
		
Parameters	β value	HR	95% CI	*p*	β value	HR	95% CI	*p*
Age	−2.250	0.105	0.020–0.550	0.002	−0.867	0.420	0.129–1.366	0.149
Gender	−0.169	0.845	0.494–1.443	0.535	−0.113	0.893	0.510–1.564	0.693
DHL	1.691	5.426	1.384–21.264	0.008	1.662	5.267	1.319–21.037	0.019
Lymphocytes	0.192	1.211	0.388–3.780	0.741	0.197	1.218	0.375–3.962	0.743
Lymphocytes (%)	−1.054	0.349	0.109–1.114	0.065	−1.258	0.284	0.088–0.921	0.036
Calcium	−0.356	0.701	0.238–2.064	0.517	−0.380	0.684	0.221–2.120	0.510
PTGG1		18.941	1.286–278.932	0.032		5.581	0.941–33.083	0.058

*DHL, Lactate dehydrogenase; HR, hazard ratio; 95% CI, Confidence Interval 95%; p-value, Cox proportional hazards estimation by partial maximum likelihood.*

Clinical features of ATL patients were stratified according to PTTG1 expression in CD4^+^ or CD8^+^ T cells. A statistically significant association between PTTG1 expression and age (HR 0.105 95%CI 0.020–0.550; *p* = 0.002), and DHL (HR 5.46; 95%CI 1.384–21.264; *p* = 0.008) was found in CD4^+^ T cells. In CD8^+^ T cells, PTTG1 expression was significantly associated with DHL concentration (HR 5.267 95%CI 1.319–21.037; *p* = 0.019) and percentage of lymphocytes (HR 0.284 95%CI 0.088–0.921; *p* = 0.036) (Table 4).

## Discussion

Herein, we have shown cell cycle arrest in G_0_/G_1_ and DNA aneuploidy in CD4^+^ T cells in HTLV-1 *in vivo*. Surprisingly, CD8^+^ T cells also showed DNA aneuploidy in HTLV-1 asymptomatic carriers and ATL patients. Moreover, the PTTG1 gene was overexpressed in ATL in both CD4^+^ and CD8^+^ T cells.

The difference in cell cycle between healthy subjects and HTLV-1 infected could be a causality of the HTLV-1 infection. Our results are in agreement with Arainga et al. (2012) who also described cell cycle arrest at G_1_ phase by human T-cell lymphotropic virus type-1 Tax protein in HeLa cells *in vitro*. Liu et al. (2008) have also showed cell cycle arrest at G_1_ phase triggered by Tax viral protein transfection and subsequent apoptosis. In the same work, the authors demonstrated the effects of HTLV-1 on HeLa cells, human osteosarcoma (HOS) cell line and T lymphoblast (SupT1). Both HTLV-1 infected HeLa cells and Tax-transduced cells exhibited high expression of anti-oncogenes p21^Cip1/WAF1^ and p27^KIP1^, mitotic abnormalities, and cell cycle arrest at G_1_. Similarly, SupT1 cells infected with HTLV-1 also revealed arrest at G_1_ phase (Liu et al., 2008).

In contrast, our study was accomplished *in vivo* using fresh lymphocytes collected from peripheral blood of HTLV-1 infected, healthy subjects and ATL patients, and were examined together for comparison purposes. Therefore, we reasonably suspected that the findings exhibited here were associated to HTLV-1. Taking this into account, we have pointed out two hypotheses to explain our data. Our first hypothesis is that the G_0_/G_1_ arrest in HTLV-1 infected might be a reaction of the infected cells against the chronic and sustained stimuli provoked by the virus. Intracellular mechanisms aiming to control and prevent replication of cells carrying genetic aberrations in genes involved in cell cycle regulation, DNA repair and apoptosis might be activated to hold cell division while DNA damage is corrected. In this hypothesis, delay of cell cycle in G_0_/G_1_ may be a step in the process of oncogenesis. However, as few HTLV-1 infected progress to ATL, the mechanisms of cell protection together with immune surveillance are usually successful and able to overcome the oncogenic stimuli. Nevertheless, when they fail, defective cells may escape immune surveillance and give rise to malignant transformation. In this hypothesis, it is reasonable to suggest that cell cycle arrest in G_0_/G_1_ may be further tested as a possible marker to identify HTLV-1 with potential to evolve to ATL. However, our results need to be further confirmed, and prospective studies with HTLV-1 infected confirming the existence of G_0_/G_1_ arrest in CD4^+^ T cells could help to elucidate the existence of a transitory event or a permanent pattern that contributes to ATL transformation. A predictive marker would be very useful, as ATL remains incurable and no biomarker for malignant transformation has been available yet (Marriott and Semmes, 2005).

In an alternative hypothesis, HTLV-1 particles and viral proteins may be interfering in the host cell cycle regulators, delaying cell division. As described above, Tax viral protein has been associated with cell cycle abnormalities such as G_0_/G_1_ delay (Liu et al., 2008; Arainga et al., 2012). On the other hand, it is possible that the virus is subverting the host biological machinery in its own benefit in order to produce proteins to supply the virus necessities. Mechanisms of cell cycle subversion have been described by many authors, as described in a review provided by Fan et al. (2018). Corroborating this explanation, Baydoun et al. (2010) showed that the protein HTLV-1 viral p30 act as a negative regulator of cell cycle delaying its progression to S-phase. These authors demonstrated that the HTLV-1 viral protein p30 binds to cyclin E and CDK2, avoiding the formation of active cyclin E-CDK2 complex, necessary for cell entry to S-phase. In the absence of the cyclin E-CDK2 complex, the Rb gene prevents E2F activation of genes required for G_1_/S transition (Baydoun et al., 2010).

Like other cancers, ATL neoplastic cells have also shown chromosomal instability and aneuploidy (Kasai et al., 2002). Similar to our findings, Kamihira et al. (1994) analyzed aneuploidy in 72 cases of ATL by flow cytometry. The authors found that 45 (62.5%) patients presented DNA aneuploidy with a DNA index ranging from 1.03 to 2.16 (mean = 1.24). They also showed an association among aneuploidy and higher tumor burden, higher level of LDH and hypercalcemia and worse survival compared with the diploid group. These authors suggested that aneuploidy evaluation should be useful to determine the clinical behavior of ATL, as well as for monitoring patient’s progress to more aggressive forms (Kamihira et al., 1994). In agreement, our data showed DNA aneuploidy in neoplastic CD4^+^ T cells in 45.0% of ATL patients, but it was unexpectedly also found in non-neoplastic CD8^+^ T cells in 25.0% of cases. Surprisingly, 42.1 and 31.6% of HTLV-1 infected exhibited DNA aneuploidy in CD4^+^ and CD8^+^ T cells, respectively. We suppose that these findings are possibly reflecting an adverse intracellular environment, caused by genetic stress due to viral particles inserted into DNA. Tax viral protein affects many targets in the cell, leading to loss of p53 checkpoint effectivity, decrease of cellular DNA repair mechanisms, such as nucleotide excision repair, base excision repair or mismatch repair, as well as checkpoint kinases 1/2, telomerase and PCNA activity (Pise-Masison et al., 2005; Matsuoka and Jeang, 2007).

Recently, Masaki et al. (2018) demonstrated that persistent and continuous exposure of Tax-CTL to HTLV-1 infected cells attenuates their function by T cell exhaustion. Likewise, we argue that DNA aneuploidy of CD8^+^ T cells found in HTLV-1 infected as well in ATL patients in our cohort could be a consequence of the chronic and continuous stimulation of HTLV-1.

Yang et al. (2011) reported that Tax triggered cyclin A, cyclin B, PTTG1, and Skp2 polyubiquitination degradation drives cells to premature entry to S-phase.

The pituitary tumor transforming gene 1 is highly expressed in normal tissues with high cell proliferation activity such as testis, thymus, embryos, and liver, while it is weakly expressed or undetectable in other normal sites (Ma et al., 2020). The high level of PTTG1 is commonly associated with an enhanced proliferative capacity, increased tumor grade and high invasive potential (Gao et al., 2019). Our data demonstrated PTTG1 gene hyperexpression in CD4^+^ T neoplastic cells of ATL, as well as in non-neoplastic CD8^+^ T cells. The cell cycle phase G_0_/G_1_ and G_2_/M were related to the expression of PTTG1 in CD4^+^ T cells and with all phases of cell cycle in CD8^+^ T cells. The PTTG1 gene is usually expressed in G_0_/G_1_, and it must be suppressed by the APC complex during mitosis to release the separase enzyme to accomplish adequate separation of the sister chromatids (Liu et al., 2003). Since PTTG1 deregulation is associated with inappropriate separation of the sister chromatids and cell aneuploidy, it may contribute for ATL tumorigenesis, given its genetic contribution for chromosome instability. In support of our study, Sheleg et al. (2007) showed that PTTG1 and Tax cooperate to increase cellular proliferation and tumorigenesis. Other studies have associated overexpression of PTTG1 and genetic instability in colorectal cancer (Kim et al., 2007), and it has been used as a prognostic marker in skin cancer (Ishitsuka et al., 2013) and breast cancer (Talvinen et al., 2008).

In this study, we did not use a second method to confirm the preliminary data of DNA ploidy. In fact, our data concerning DNA ploidy and cell cycle was obtained by flow cytometry. Nevertheless, this technique has been widely used in scientific literature to study cell cycle and measure DNA ploidy. In addition, we used reagents previously validated and internal and external controls as recommended. Therefore, the proceedings were carried out in equipment and instruments previously calibrated and using specific software developed and standardized for cell cycle analysis. Moreover, the proceedings were taken according to the convention on nomenclature for DNA cytometry (Hiddemann et al., 1984). According to this, the DNA measurement by cytometric analysis must be differentiated from that provided by cytogenetic techniques. Thus, to emphasize the difference between DNA aneuploidy and “true” aneuploidy derived from karyotype analysis, the prefix “DNA” must be used before the term aneuploidy. In this consensus, the presence of at least two separate G_0_/G_1_ peaks can be interpreted as “true” aneuploidy. As also described by the authors, the coefficient of variation of the G_0_/G_1_ peak of the analyzed cells was monitored closely throughout the proceedings (Shankey et al., 1993). By using this approach, we undoubtedly showed abnormal DNA ploidy in CD4^+^ and CD8^+^ T cells in HTLV-1 infected and ATL. In fact, the agreement of aneuploidy results obtained between flow cytometry and karyotype method is higher using fresh samples, which was the case in this study, instead of paraffin-embedded tissues (Almozain et al., 2018).

In conclusion, we demonstrated an increase of cells in G_0_/G_1_ phase in CD4^+^ T cells in HTLV-1 asymptomatic carriers, and higher S-phase in ATL patients. Unexpectedly, CD8^+^ T cells in ATL patients showed increment in the S-phase. DNA aneuploidy was found in CD4^+^ and CD8^+^ T cells of HTLV-1 asymptomatic carriers and ATL. Moreover, PTTG1 gene was overexpressed in CD4^+^ and CD8^+^ T cells of ATL patients.

## Data Availability Statement

The raw data supporting the conclusions of this article will be made available by the authors, without undue reservation, to any qualified researcher.

## Ethics Statement

The studies involving human participants were reviewed and approved by the CAPPesq HC-FMUSP number 814/08. The patients/participants provided their written informed consent to participate in this study.

## Author Contributions

DL, SB, and JP: conception and design. DL, GB, and JP: development of methodology. MF, GB, LL, and HC: acquisition of data (acquired and managed patients). DL, JP, and CR: analysis and interpretation of data (e.g., statistical analysis, biostatistics, computational analysis). DL, LL, HC, YN, and JP: writing, review, and revision of the manuscript. MF, DL, GB, and LA: administrative, technical, or material support (i.e., reporting or organizing data, constructing databases). JP: study supervision. All authors contributed to the article and approved the submitted version.

## Conflict of Interest

The authors declare that the research was conducted in the absence of any commercial or financial relationships that could be construed as a potential conflict of interest.
